# In vivo analysis of covering materials composed of biodegradable polymers enriched with flax fibers

**DOI:** 10.1186/s40824-017-0094-6

**Published:** 2017-05-19

**Authors:** Tomasz Gredes, Sandra Schönitz, Tomasz Gedrange, Lukas Stepien, Karol Kozak, Christiane Kunert-Keil

**Affiliations:** 1 Department of Orthodontics, Carl Gustav Carus Campus, Technische Universität Dresden, Fetscherstr. 74, D-01307 Dresden, Germany; 2 Clinic for Neurology, Carl Gustav Carus Campus, Technische Universität Dresden, Fetscherstr. 74, D-01307 Dresden, Germany; 3 Fraunhofer IWS, Winterbergstr. 28, D-01277 Dresden, Germany

**Keywords:** Flax composites, PLA, PCL, Biocompatibility, Inflammation

## Abstract

**Background:**

The objective of this study was to investigate the in vivo effect of bioactive composites with poly(lactic acid) (PLA) or polycaprolactone (PCL) as the matrix, reinforced with bioplastic flax fibers, on the surrounding muscle tissue.

**Methods:**

Materials of pure PLA and PCL and their composites with flax fibers from genetically modified plants producing poly-3-hydroxybutyrate (PLA-transgen, PCL-transgen) and unmodified plants (PLA-wt, PCL-wt) were placed subcutaneous on the M. latissimus dorsi for four weeks.

**Results:**

The analysis of histological samples revealed that every tested material was differently encapsulated and the capsule thickness is much more pronounced when using the PCL composites in comparison with the PLA composites. The encapsulation by connective tissue was significantly reduced around PCL-transgen and significantly increased in the cases of PLA-transgen and PLA-wt. In the collected muscle samples, the measured protein expression of CD45, lymphocyte common antigen, was significantly increased after the use of all tested materials, with the exception of pure PCL. In contrast, the protein expression of caveolin-1 remained unchanged after treatment with the most examined materials. Only after insertion of PLA-wt, a significant increase of caveolin-1 protein expression was detected, due to the improved neovascularization.

**Conclusion:**

These data support the presumption that the new bioactive composites are biocompatible and they could be applicable in the medical field to support the regenerative processes.

## Background

Large bone defects often require bone-graft implantation to promote bone regeneration. Although autograft remains the gold standard for bone repair, the harvesting of autologous bone is limited and for some patients the usage can even be associated with complications [1]. The use of allografts, as an alternative, is not always a satisfactory solution for bone substitution because of increased risks of pathogen transmissions, poor healing capacity, and adverse immune reactions [2]. For that reason, new strategies for bone healing are demanding by means of natural or synthetic materials. Synthetic bone graft substitutes should be biocompatible, cause minimal fibrotic reaction, support new bone formation, undergo remodeling processes, and ideally, they should have similar strength to that of the cortical/cancellous bone being replaced [3].

Biodegradable synthetic polymers, such as polycaprolactones [PCL; (1,7)-polyoxepan-2-one], poly(lactid acid) (PLA), polyanhydrides (PAHs), poly-3-hydroxybutyrate (P3HB; 3-hydroxybutanoic acid), poly(propylene fumarate), poly(glycolic acid) (PGA), and associated copolymers are interesting for wide use as matrix or components for scaffolds in tissue engineering and regenerative medicine, due to their various mechanical and physical properties, degradation times, or modes of degradation [4, 5].

Numerous studies have shown the usefulness of P3HB for bone regeneration, and they have also indicated that the degradation products of P3HB are phagocytosed by macrophages and osteoclasts; they show no toxicity and may even stimulate the osteoblasts in a positive manner [6, 7]. P3HB scaffolds are not only highly compatible with osteoblast but they also can induce ectopic bone formation [8]. Concerning the preparation of biomaterials for tissue repair, PLA and PGA homo- and copolymer were, for a long time, the first choice compared with other biodegradable polymers [9]. The excreted products during degradation of PLA occur in cell metabolism of all microorganisms and animals incorporated into the tricarboxylic acid cycle and they are assumed to be completely non-toxic [10]. Items produced from PLA have already been clinically used with good results, inter alia, for craniofacial fracture and ankle fixation [11]. Disadvantages of these materials are the stiffness and plastic deformation characteristics which have limited their applications. In comparison to the PLA, PCL is a relatively flexible and very slow degradable biomaterial [12]. PCL is used in several biomedical applications such as wound dressings, fixation devices or scaffolds for bone and cartilage tissue engineering and drug delivery devices [10, 13, 14].

In order to achieve better mechanical properties, similar to bone tissue, polymer composite materials can be reinforced with natural fibers. It has been shown that the application of such composites as medical devices was beneficial regarding the biocompatibility in the human body compared to single materials such as polymers, ceramics and metals [15].

Natural fibers have the advantage that they are renewable resources and have marketing appeal. It could be proven that some natural fibers prevent stress shielding and increase bone remodeling [16, 17]. Furthermore, many studies have shown that flax fibers have better mechanical properties, similar to glass fibers, and lower water absorption, in comparison with other natural fibers [18–20]. Recently, a hybrid natural fiber polymer composite material based on epoxy resin, enriched with sisal, jute and hemp fibers, was proposed for clinical application as orthopedic implants for femur bone prosthesis because of its good material properties, such as low density, increased tensile, compression, bending strength, and the mechanical behavior comparable to that of long human bones [17, 21]. In recent years, a new generation of transgenic plants was increasingly developed for the production of recombinant medicines and industrial products, such as vaccines, antibodies, biofuels and plastics [22–25]. For instance, a newly developed dressing of genetically modified flax fibers producing antioxidants was used for chronic wound treatment with good results [26].

It has been shown that P3HB can be successfully synthesized in transgenic plants such as cotton, tobacco, potato or flax [27, 28]. In nature, P3HB is synthesized as carbon sources and electron sink by a large number of microorganisms, including gram-negative, gram-positive aerobic and photosynthetic species, lithotrophs or organotrophs as inclusion bodies to serve energy [29].

Recently, flax plants were transformed with three bacterial genes responsible for the synthesis of P3HB [30]. It could be shown that the flax fibers expressing P3HB exhibited more favorable mechanical properties than unmodified flax fibers [31]. The fibers isolated from transgenic flax plants showed better biomechanical properties compared to native flax plants. The measured parameters such as, strength, Young’s modulus, and energy for failure of flax fibers, were significantly increased. Thereby, improved elastic properties of fibers from the transgenic line and theirs composites could be demonstrated [32]. A previous study showed that a composite made of polypropylene and fibres with P3HB from transgenetic plants had a lower maximum tensile strength than pure polymer. The material with fibers from transgenic plants was significantly stronger and it showed a higher Young’s modulus than the composites prepared with native flax fibres. This indicated a stronger bond of the transgenic flax fibres to the matrix than fibers from the control flax plants [33].

Using these transgenic bioplastic flax fibers embedded in PLA or PCL matrix, a new generation of biodegradable and bioactive composites was prepared and initially analyzed [30]. A good biocompatibility of these new materials has already been proven in previous in vitro experiments [34]. We could show that composites with transgenic flax fibers had a positive effect on bone regeneration which occurred faster than after treatment with composites with non-transgenic flax plants [35]. Due to their bacteriostatic and platelet anti-aggregated properties, the new flax composites were suggested as a new source of material for medical applications.

The question was whether the enrichment of polyesters with flax fibers affected intensified inflammatory reaction in another tissue than bone. In a previous study, we could not detect any enhanced inflammation after insertion these materials on the cranial bone of rat [36].Thus, the objective of this study was the investigation of the influence of the bioactive composites on the surrounding muscle and connective tissue.

## Methods

### Composites

For the preparation of the composites, two kinds of matrix, PLA and PCL, obtained from Biomer (Germany), were used. The polymers were reinforced with a 20% content of flax fibers (either wt- unmodified flax fibers or transgenic flax fibers which contained P3HB). Pulverized combed flax fibers were mixed with PLA or PCL granules at 170°C. The mixture was mechanically pressed into sheets. These were cut into smaller pieces and heat-pressed between Teflon sheets, 30–60s at 175°C for the PLA composite or 100°C for the PCL composite, to produce final composite sheets of 0.2 mm thickness [30, 34, 35, 37].

A previous study of prepared composites on mechanical analysis showed improved stiffness and a decrease in tensile strength. Furthermore, bacteriostatic and platelet anti-aggregated properties of these new materials could be proven [30].

### Surgical procedure and experimental design

Experiments were performed on 2-month old Lewis 1A rats of both sexes (*n* = 44). The approvals for all surgical and experimental procedures were issued by the Animal Welfare Committee of the State Government of Mecklenburg-Western Pomerania (LALLF M-V/TSD/7221.3-1.1-094/11). The surgical procedure was performed using a fixed protocol established by Gredes et al., 2010 [38].

Following test materials were applied in subcutaneous pockets on the back of rats:

- group 1 (*n* = 6; PLA) pure PLA
- group 2 (*n* = 6; PLA-transgen) biocomposite of PLA and flax fibers containing P3HB
- group 3 (*n* = 6; PLA-wt) biocomposite of PLA and unmodified flax fibers
- group 4 (*n* = 7; PCL) pure PCL
- group 5 (*n* = 7; PCL-transgen) biocomposite of PCL and flax fibers containing P3HB
- group 6 (*n* = 7; PCL-wt) biocomposite of PCL and unmodified flax fibers
- group 7 (*n* = 5; control) animals without any treatment.

After four weeks, all animals were killed with an overdose of isoflurane and the composites with the surrounding muscle tissue of the Musculus latissimus dorsi were harvested and subjected to histological analysis.

#### Histological analysis

For histological examination, muscle samples passed the standard protocol for embedding in paraffin. Afterwards, the paraffin blocks were trimmed in serial longitudinal sections of about 3 μm at a microtome (Leica RM 2155). The sections were stained at first with hematoxylin/eosin (H.E.) for the assessment of the encapsulation of test materials in surrounding soft tissue. The evaluation of the granulo-fibrous connective tissue capsule around the composites and division in grades was done as described by Miura et al. [39].

For immunohistochemical staining, antibodies against CD45 (lymphocyte common antigen) as well as caveolin-1 (expressed in endothelial cells) were used. For that, the specimens were firstly deparaffinized, rehydrated, rinsed for 10 minutes in tris-buffered saline (TBS) and incubated in citrate buffer in a water bath at 95–99°C for 40 minutes. The slides were cooled down for 20 minutes at room temperature. In order to remove endogenous peroxidase, methanol and 3% H_2_O_2_ were used for 10 minutes. After retrieval of specimens, they were rinsed with distilled water and TBS and thereafter incubated with the primary antibodies in a humid chamber (rat monoclonal anti-CD45 diluted 1:50; BD Biosciences, Heidelberg, Germany or mouse monoclonal anti-caveolin 1; clon 7C8, 2.5 μg/ml; LifeSpan BioSciences, Seattle, USA). The visualization of the bound caveolin-1 and CD45 antibodies was done using a New Fuchsine alkaline phosphatase substrate protocol. All sections were then lightly counterstained with hematoxylin and cover-slipped. For negative controls the primary antibody was replaced by PBS. The determination of antigen level was carried out using a blind test, which was conducted at the same time with identical staff, equipment and chemicals. For quantification of the protein expression levels, 5–10 digital pictures with a magnification of x200 were randomly taken in different areas of each section (Zeiss light microscope, Axio Scope A1 equipped with a digital camera AxioCam MRc, Zeiss, Jena, Germany). For all sections, the quantity of pixels that had a positive reaction for cav-1 and CD45 was assessed using cell^F Ink software (analySIS Image Processing Olympus, Münster) as described by Kunert-Keil et al., [40].

#### Statistical analysis

All data were analyzed using SigmaPlot software (Systat Version 3.5 Software, Inc., San Jose, CA, USA) and statistically analyzed using Student’s unpaired t-test (measurements with normal distribution) or Mann-Withney-U rank sum test (measurements without normal distribution). Data are shown as mean ± SEM, and *p <* 0.05 was set as the level of statistical significance.

## Results

### Encapsulation of the composites

**Fig. 1:**
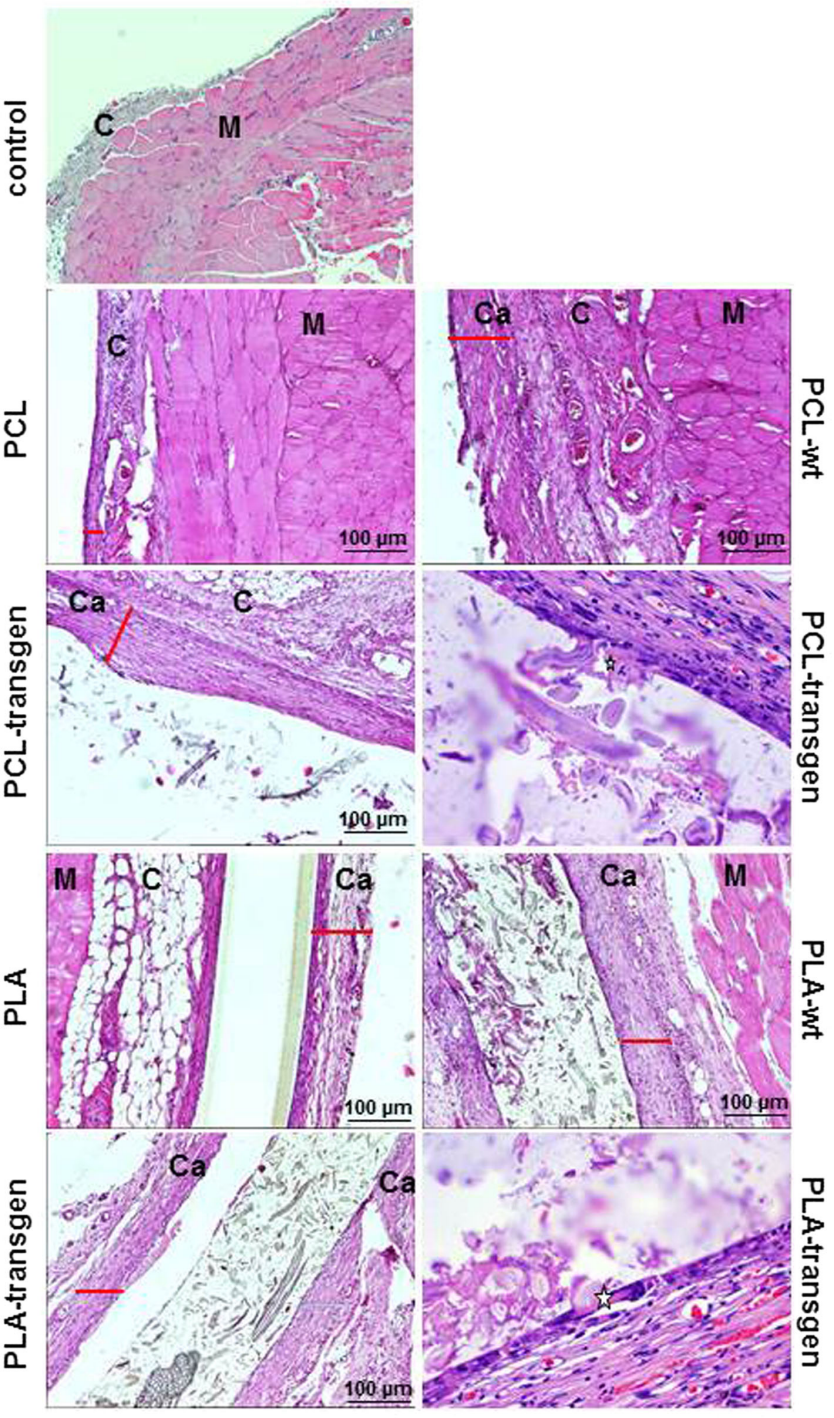
Reaction of the Musculus latissimus dorsi to the different flax composite. Sample images of connective tissue capsules between muscle and material stained with hematoxylin/eosin. Ca = capsule; M = muscle; C = connective tissue; star = giant cell; the red line shows the thickness of the capsule

**Fig. 2:**
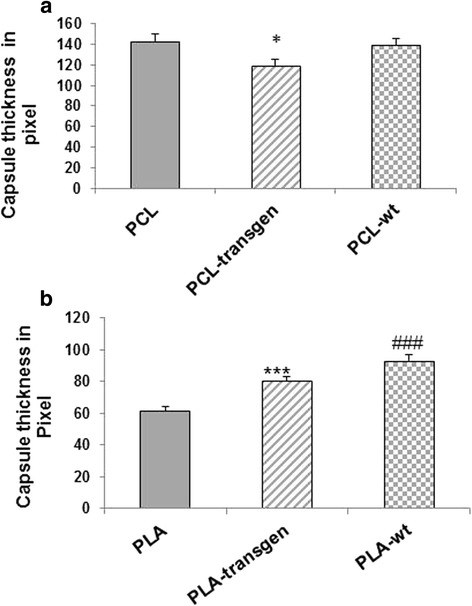
Quantification of the encapsulation of the (**a**) PCL and (**b**) PLA composites using CellF software. Indicated are mean ± standard error of *n* = 6–7 single preparations. Mann-Whitney U rank sum test; *P < 0.05 indicates statistically significant differences between PCL and PCL-transgen; ***P < 0.005 indicates statistically significant differences between PLA and PLA-transgen; ###*P <* 0.005 indicates statistically significant differences between PLA and PLA-wt

Histological specimens showed 4 weeks after insertion of the test materials a well-vascularized connective tissue between the back muscle and the inserted materials. Furthermore, the inserted materials were covered by a compact capsular structure composed of granulo-fibrous connective tissue. This tissue was classified as grade 4, fibrous connective tissue with few cellular components aligned in parallel. Fig. 1 showed exemplary the expression of the collagen capsule with the pure polymers as well as the different flax composites. In addition, giant cells are presented at the surface of the composites. In controls, these compact capsular structure could not be detected (Fig. 1). The capsule thickness served as an indicator of the biocompatibility of the materials in the body. The quantitative analysis of the capsule thickness resulted in a capsule width between 60–142 pixels (Fig. 2). It has been found that the capsule thickness is much more pronounced when using the PCL composites, in comparison with the PLA composites. The use of PCL-transgen led compared to pure PCL to a significant reduction of the capsule thickness by 15%, whereas no change in the capsule thickness was observed by use of PCL-wt. The usage of PLA-transgen resulted in a 1.3 fold increase in capsule thickness compared to pure PLA. Furthermore, the use of wild-type flax was followed by repeated widening of the fibrous capsule (Fig. 2).

### Expression of CD45

**Fig. 3:**
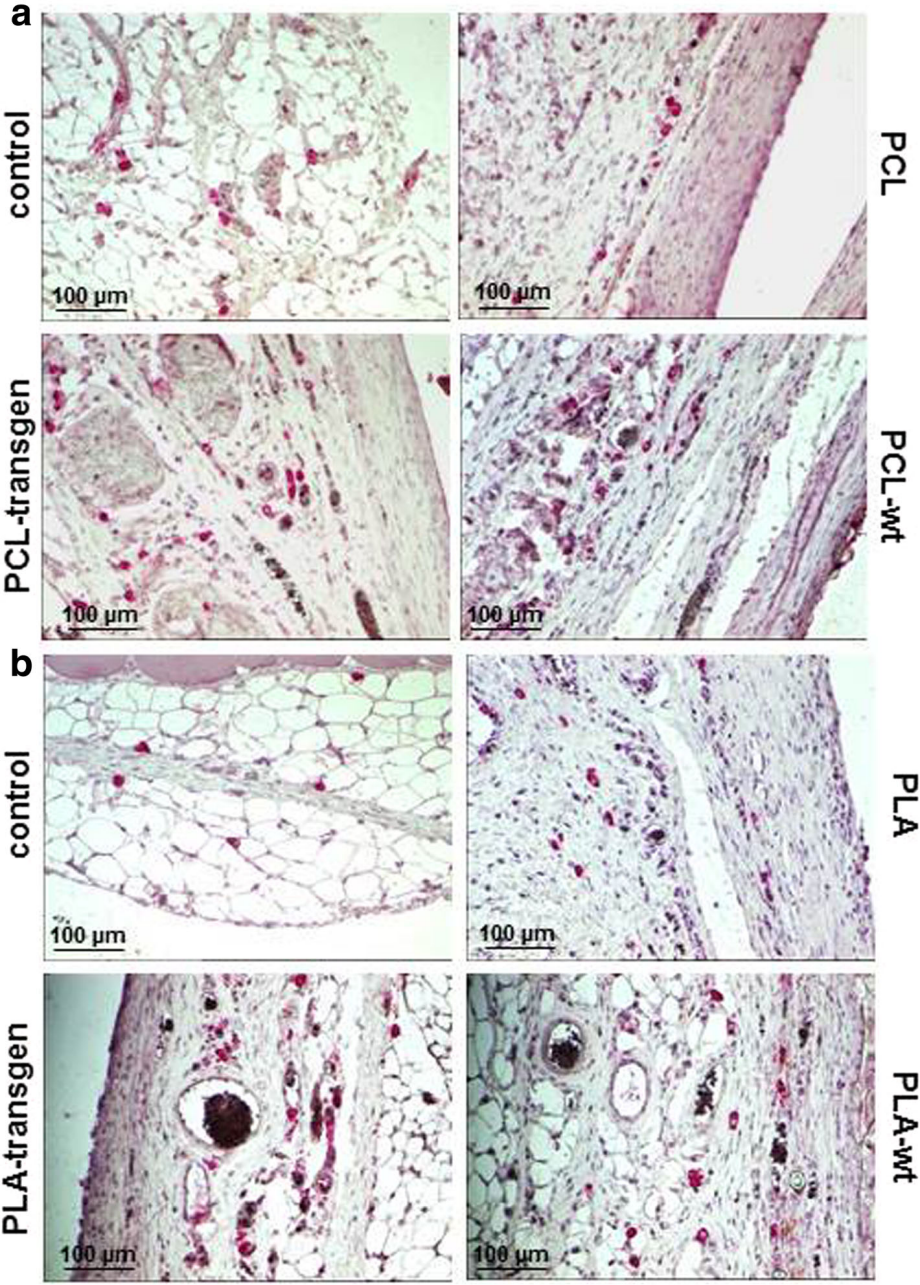
Immunohistological staining of CD45 in the tissue specimens four weeks after insertion of the different (**a**) PCL and (**b**) PLA composites

**Fig. 4:**
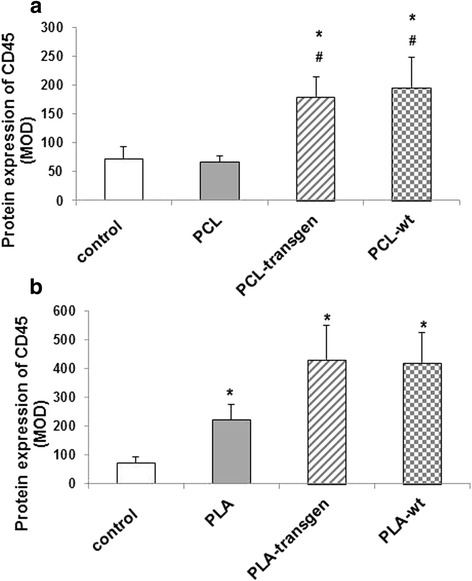
Quantification of the CD45 expression using the software CellF after insertion of the different (**a**) PCL and (**b**) PLA composites. Indicated are mean ± standard error of *n* = 6–7 single preparations. Mann-Whitney U rank sum test; *P < 0.05 indicates statistically significant differences between control and composite; #*P <* 0.05 indicates statistically significant differences between PCL and flax composite

CD45 positive lymphocytes were immunohistochemical stained in the form of red precipitates on the histological specimens and are only visible in the loose connective tissue and not in the capsule (Fig. 3). The quantitative analysis of the expression of CD45 resulted in the following data:

1. Pure PCL did not induce inflammatory response.
2. When using PCL-flax composites there was a significant increase in the number of CD45 positive cells by 2.5–3fold compared to both, control as well as pure PCL.
3. There is no difference between the composites of transgenic and wild-type flax.
4. In the tissue between pure PLA and the muscle, the evaluation of the expression of CD45 showed a significant increase of CD45 positive lymphocytes to 3.1 times. When using the PLA-flax composites the number of inflammatory cells increased significantly compared to the pure polymer again by about 50% to (mean ± standard error, control vs. PLA; 73 ± 21 vs. 223 ± 52, *P* = 0.037; control vs. PLA transgene: 73 ± 21 vs. 433 ± 117, *P* = 0.023; control vs. PLA wt: 73 ± 21 vs. 421 ± 104, *P* = 0.017; Fig. 4).

### Expression of caveolin-1

**Fig. 5:**
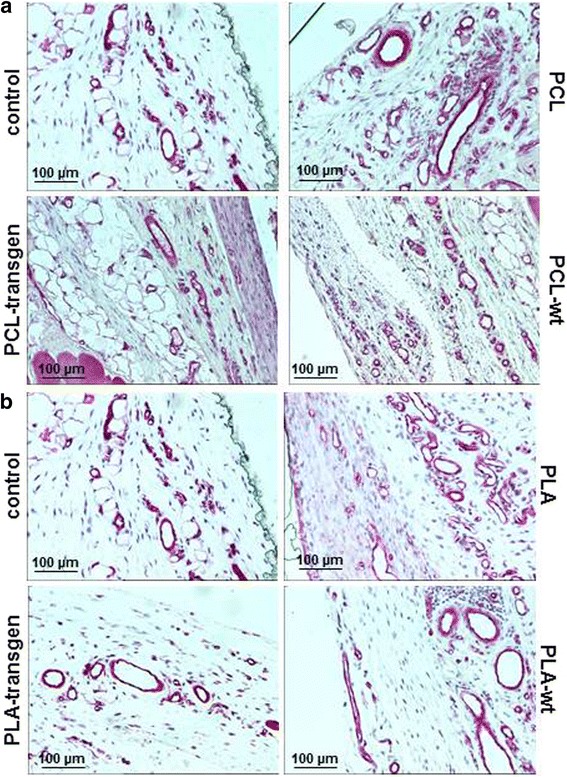
Immunohistological staining of caveolin-1 in the tissue specimens four weeks after insertion of the different (**a**) PCL and (**b**) PLA composites

Figure 5 showed an example of the immunohistochemical detection of caveolin-1 in the different study groups.

**Fig. 6:**
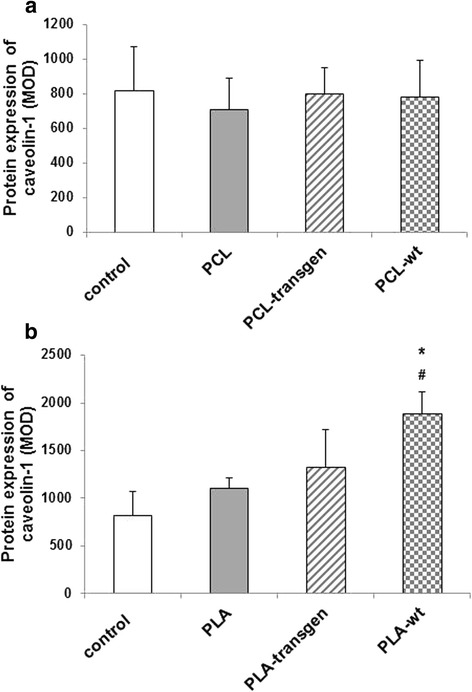
Quantification of the caveolin-1 expression using CellF software after insertion of the different (**a**) PCL and (**b**) PLA composites. Indicated are mean ± standard error of *n* = 6–7 single preparations. Mann-Whitney U rank sum test; *P < 0.05 indicates statistically significant differences between control and PLA-wt; #*P <* 0.05 indicates statistically significant differences between PLA and PLA-wt

After insertion of the tested PLA-wt composites blood flow of the surrounding muscle tissue was positively influenced (Fig. 5). Compared to control and PLA-treated animals there was a significant increase in caveolin-1 expression between 170–230% (mean ± standard error; control vs. PLA-wt: 817 ± 258 vs. 1882 ± 237, *P* = 0.014; PLA vs. PLA-wt: 1103 ± 116 vs. 1882 ± 237, *P* = 0.043; Fig. 6). In contrast to the PLA composites, PCL composites do not induce or reduce the vascularization (Fig. 6).

## Discussion

The biocompatibility of polymer-flax composites was examined in the rat model after their positioning on M. latissimus dorsi. The analysis included the morphometric determination of the capsule thickness, the amount of CD45 positive cells and the formation of new blood vessels in the contact area of the tested materials with the surrounding muscle tissue.

The macroscopic evaluation of muscle samples has not demonstrated any inflammatory reactions, neither after treatment with flax composites nor pure polymers [38]. As described in the literature, flax fibers have in vivo good biocompatibility, they are allergen free and not carcinogenic [30, 41–43]. A further study showed that an application of a modified flax-dressing bandage could lead to a more rapid rate of healing and reduce the wound exudes and wound size, thanks to the hygroscopic properties of the flax fibers which ensure the optimal humidity for the ulcer, absorb the excess of exudates and reduce the inflammation in the wound [44].

Our investigations revealed slight inflammatory reactions in muscle tissue after subcutaneous implantation of all materials. However, when considering solely pure polymers, a significant increase of CD45 positive cells was only observed in the muscle after treatment with PLA, while the protein expression of CD45 after PCL application remained constant. Surprisingly, the histological specimens have shown that PLA flax composites 4 weeks after insertion were increasingly encapsulated in comparison to the pure polymer. Furthermore, the polymers after four weeks were encapsulated, wherein the capsule after treatment with PCL was 2.5 times thicker than that of PLA. After addition of flax components significantly increased foreign body reaction was found in case of PLA flax composites, which was reflected in thicker encapsulation and thereby stronger isolation from the host tissues. In contrast, the thickness of capsule around PCL flax composites remained unchanged (PCL-wt) or were significantly reduced (PCL-transgen) compared to pure PCL. It is well known, that encapsulation around implants generally occurs as a result of wound healing and foreign body reaction [39, 45]. As described by Miura et al. the capsule can be divided into four grades and the histopathological scores significantly decrease with time independently of the implantation site [39]. A correlation between vascularization and capsule formation was controversially discussed in the literature. We have found that both materials had no effect on the vascularization in the surrounding tissue. Park et al. demonstrated no differences for both, vascularity and CD34 expression for two different silicone implants, whereas the capsule thickness was significantly different after 4 and 12 weeks [46]. The authors found that a stronger foreign body reaction leads to more excessive capsular formation. Furthermore, it was postulated that from the capsule thickness and density, the foreign body reactions against implanted materials can be concluded [46]. This means a material with better biocompatibility produces a thin capsule. In case of our study, it would mean that PLA composites are better compatible than PCL composites. However, all PLA scaffolds had a stronger expression of CD45 positive cells compared to PCL composites.

Despite the good biocompatibility and the medical usage of PLA [41, 43, 47], some in vivo studies showed inflammatory reactions induced by degradation of this polymer [48, 49]. However, these inflammatory processes had no impact on the neovascularization in the surrounding tissue and it could previously be detected that the neovascularization was correlated with more orderly deposition of collagen [48]. Our study showed similar results. Although the expression of CD45 was increased, the expression of caveolin-1 remained mainly stable. A significant increase of caveolin-1 protein expression was detected only after insertion of PLA-wt, in correlation with the improved neovascularization. It has been already shown that caveolin-1 expression appears to be correlated with progressive stages of the angiogenic process: caveolin-1 is strongly abundant in endothelial cells regulating important functions as angiogenesis, vascular permeability, and transcytosis [50]. In a recent study on caveolin 1-deficient mice, angiogenesis was found to be markedly reduced in comparison with control mice [51].

The encapsulation of PLA, accompanied by macrophages and giant cells, could be detected after subcutaneous implantation [52]. Similar histological findings of encapsulation were demonstrated after intraperitoneal injection of PLA particles [53]. It is assumed that the degradation products and the rate of degradation may play an important role in determining the local cellular response to the implanted material. It has already been shown, that both tested polymers are biodegradable and they decompose during hydrolysis into monomers and other degradation products, which play an important role regarding the biocompatibility [54]. PLA degrades inter alia, to lactic acid, which is further metabolized either in the citric acid cycle or excreted through the kidneys [55]. The hydrolytic degradation of PLA depends on its molecular weight, the polydispersity, crystallinity and its form [56–58]. This could be shown in some in vivo long-term animal studies after implantation of PLA into the bone tissue [11, 59]. While PLA implants with higher molecular weight and higher crystallinity were not completely absorbed after 6 years [60], the other implants with lower crystallinity or made of amorphous PLA were fully resorbed and undetectable after 1 to 3 years [61]. Further studies in a rat model have shown that amorphous PLA implants were completely degraded even within 39 weeks without chronic inflammatory responses [43, 47].

In addition, a correlation between the degradation of the polymers and the capsule thickness could be observed in many studies. Grayson et al. proved that the fibrous capsules surrounding implants made of polymers, showed greater maturation of the capsules for the more rapidly degrading materials after 21 days (e.g. PLGA), but less mature capsules was observed in case of PLA up to 49 days [62]. Qu et al. observed much slower degradation of pure P3HB compared to P3HB composites, e.g. P3HB-co-3-hydroxyhexanoate (PHBHHX) and PLA. The connective tissue response after PHB insertion was much more pronounced than after treatment with PLA and PHBHHx [63]. It is known that polyesters such as PGA, PLA and PCL are degraded basically by a non-enzymatic random hydrolytic scission of esters linkage with different rate. For comparison, PGA degrades fast, PLA slow and PCL very slow depending on the hydrophilicity of each monomeric unit [64]. Hence, the much slower degradation of PCL in contrast to other biodegradable polymers could also explain the stronger encapsulating of PCL compared to PLA.

In vivo degradation of PCL can be divided into two phases. The first long-term phase is a superficial non-enzymatic hydrolytic cleavage of the ester groups without loss of quantity and deformation of the material. In the subsequent second phase, the molecular weight as well as the volume of polymer significantly decreases and released small particles are further degraded during phagocytosis, and finally excreted [10, 65]. A long term study in rats demonstrated that the molecular weight (Mw) of PCL deceased over time and followed a linear relationship between logMw and time. PCL broke into low molecular weight pieces at the end of 30 months. PCL capsules remained intact in shape during 2-year implantation and after degeneration the material could be completely excreted and did not cumulate in body tissue [66].

A subcutaneous implantation of PCL in the rabbit model was well tolerated and resulted after 6 months in only 1% of mass loss of this polymer. No inflammatory reactions or other pathological effects in the surrounding tissue could be histologically detected neither after 3 nor after 6 months [67]. Ultrathin membranes of PCL tested in rats and pigs for a skin wound covering also did not cause any inflammatory responses in surrounding tissue and had a positive impact on the skin regeneration processes [68]. Furthermore, a good compatibility of PCL plugs was shown after their insertion in the skull with a direct contact with the brain. The results have indicated that PCL did not evoke any undesirable inflammatory response and additionally, neurogenic potential was not negatively impacted [69]. Thought, postoperative ocular inflammation was seen in 67% of eyes for 1 week, the histological analysis revealed no ocular inflammation or morphologic abnormalities of ocular tissues and no cellular reaction, fibrosis, or surface biodeposits. Thin-film micro- and nanostructured PCL appeared to be a feasible biomaterial for intraocular therapeutic applications [70].

Due to their biodegradability and good biocompatibility, both PLA and PCL were also used pure or as copolymer, in order to ameliorate the acidic byproducts, for many new scaffolds in the regenerative medicine which are still of increased interest.

## Conclusions

This study reports on the suitability and effectiveness of the biomaterials reinforced with flax fibers in the field of regenerative medicine. Although these materials, according to the macroscopic analysis, did not cause any necrotic processes in the surrounding tissue, immunohistological examinations revealed that the expression of inflammatory cells increased significantly compared to the pure polymer. Furthermore, the physical and mechanical properties of the new materials could be improved for their better application and handling.

## Abbreviations

H.E.: Haematoxylin/eosin
Mw: Molecular weight
P3HB: Poly-3-hydroxybutyrate
P3HBHHX: P3HB-co-3-hydroxyhexanoate
PAHs: Polyanhydrides
PCL: Polycaprolactone
PGA: Poly(glycolic acid)
PLA: Poly(lactide acid)
PLGA: Poly(lactic-co-glycolic acid)
TBS: Tris-buffered saline
Wt: Wildtype (genetically unmodified plant)

## References

[1] Sen MK, Miclau T (2007). Autologous iliac crest bone graft: should it still be the gold standard for treating nonunions?. Injury..

[2] Agarwal R, Garcia AJ (2015). Biomaterial strategies for engineering implants for enhanced osseointegration and bone repair. Adv Drug Deliv Rev..

[3] Giannoudis PV, Dinopoulos H, Tsiridis E (2005). Bone substitutes: an update. Injury..

[4] Matassi F, Nistri L, Chicon Paez D, Innocenti M (2011). New biomaterials for bone regeneration. Clin Cases Miner Bone Metab.

[5] Misra SK, Ansari TI, Valappil SP, Mohn D, Philip SE, Stark WJ (2010). Poly(3-hydroxybutyrate) multifunctional composite scaffolds for tissue engineering applications. Biomaterials.

[6] Kikuchi M, Tanaka J, Koyama Y, Takakuda K (1999). Cell culture test of TCP/CPLA composite. J Biomed Mater Res.

[7] Saad B, Ciardelli G, Matter S, Welti M, Uhlschmid GK, Neuenschwander P (1998). Degradable and highly porous polyesterurethane foam as biomaterial: effects and phagocytosis of degradation products in osteoblasts. J Biomed Mater Res.

[8] Mai R, Hagedorn MG, Gelinsky M, Werner C, Turhani D, Spath H (2006). Ectopic bone formation in nude rats using human osteoblasts seeded poly(3)hydroxybutyrate embroidery and hydroxyapatite-collagen tapes constructs. J Craniomaxillofac Surg..

[9] Lu W, Ji K, Kirkham J, Yan Y, Boccaccini AR, Kellett M (2014). Bone tissue engineering by using a combination of polymer/Bioglass composites with human adipose-derived stem cells. Cell Tissue Res.

[10] Pitt CG, Gratzl MM, Kimmel GL, Surles J, Schindler A (1981). Aliphatic polyesters II. The degradation of poly (DL-lactide), poly (epsilon-caprolactone), and their copolymers in vivo. Biomaterials.

[11] Athanasiou KA, Agrawal CM, Barber FA, Burkhart SS (1998). Orthopaedic applications for PLA-PGA biodegradable polymers. Arthroscopy.

[12] Shi R, Chen D, Liu Q, Wu Y, Xu X, Zhang L (2009). Recent advances in synthetic bioelastomers. Int J Mol Sci.

[13] Bayati V, Abbaspour MR, Dehbashi FN, Neisi N, Hashemitabar M. A dermal equivalent developed from adipose-derived stem cells and electrospun polycaprolactone matrix: an in vitro and in vivo study. Anat Sci Int. 2016; 10.1007/s12565-016-0352-z 27329656

[14] Hutmacher DW (2000). Scaffolds in tissue engineering bone and cartilage. Biomaterials.

[15] Ramakrishna S, Huang ZM, Kumar GV, Batchelor AW, Mayer J (2004). An Introduction to biocomposites.

[16] Gouda TA, Jagadish SP, Dinesh KR, Gouda H, Prashanth N (2014). Characterization and investigation of mechanical properties of hybrid natural fiber polymer composite materials used as orthopaedic implants for femur bone prosthesis. IOSR-JMCE.

[17] Heary RF, Parvathreddy NK, Qayumi ZS, Ali NS, Agarwal N (2016). Suitability of carbon fiber-reinforced polyetheretherketone cages for use as anterior struts following corpectomy. J Neurosurg Spine.

[18] Frederick TW, Norman W (2004). Natural fibers plastics and composites.

[19] Mohanty AK, Misra M, Hinrichsen G (2000). Biofibers, biodegradable polymers and biocomposites: an overview. Macromol Mater Eng.

[20] Satyanarayana KG, Wypych F, Bhattacharyya D, Fakirov S (2007). Characterization of natural fibers. Engineering biopolymers: homopolymers, blends and composites.

[21] Chandramohan D, Marimuthu K (2011). Characterization of natural fibers and their application in bone grafting substitutes. Acta Bioeng Biomech.

[22] Conrad U (2005). Polymers from plants to develop biodegradable plastics. Trends Plant Sci.

[23] Key S, Ma JK, Drake PM (2008). Genetically modified plants and human health. J R Soc Med.

[24] Ma JK, Drake PM, Christou P (2003). The production of recombinant pharmaceutical proteins in plants. Nat Rev Genet.

[25] Sticklen M (2006). Plant genetic engineering to improve biomass characteristics for biofuels. Curr Opin Biotechnol.

[26] Skorkowska-Telichowska K, Kulma A, Szopa J (2012). The response of diabetic foot to a new type of dressing. Int Arch Med.

[27] John ME, Keller G (1996). Metabolic pathway engineering in cotton: biosynthesis of polyhydroxybutyrate in fiber cells. Proc Natl Acad Sci U S A.

[28] Somleva MN, Peoples OP, Snell KD (2013). PHA bioplastics, biochemicals, and energy from crops. Plant Biotechnol J.

[29] Bonartsev AP, Bonartseva GA, Shaitan KV, Kirpichnikov MP (2011). Poly(3-hydroxybutyrate) and poly(3-hydroxybutyrate)-based biopolymer systems. Biochem Supp Series B Biomed Chem.

[30] Wrobel-Kwiatkowska M, Czemplik M, Kulma A, Zuk M, Kaczmar J, Dyminska L (2012). New biocomposites based on bioplastic flax fibers and biodegradable polymers. Biotechnol Prog.

[31] Wrobel M, Zebrowski J, Szopa J (2004). Polyhydroxybutyrate synthesis in transgenic flax. J Biotechnol.

[32] Wrobel-Kwiatkowska M, Zebrowski J, Starzycki M, Oszmianski J, Szopa J (2007). Engineering of PHB synthesis causes improved elastic properties of flax fibers. Biotechnol Prog.

[33] Szopa J, Wrobel-Kwiatkowska M, Kulma A, Zuk M, Skorkowska-Telichowska K, Dyminska L (2009). Chemical composition and molecular structure of fibers from transgenic flax producing polyhydroxybutyrate, and mechanical properties and platelet aggregation of composite materials containing these fibers. Composites Science and Technology.

[34] Kunert-Keil C, Gredes T, Meyer A, Wrobel-Kwiatkowska M, Dominiak M, Gedrange T (2012). The survival and proliferation of fibroblasts on biocomposites containing genetically modified flax fibers: an in vitro study. Ann Anat.

[35] Gredes T, Wrobel-Kwiatkowska M, Dominiak M, Gedrange T, Kunert-Keil C (2012). Osteogenic capacity of transgenic flax scaffolds. Biomed Tech (Berl).

[36] Gredes T, Kunath F, Gedrange T, Kunert-Keil C (2016). Bone Regeneration after Treatment with Covering Materials Composed of Flax Fibers and Biodegradable Plastics: A Histological Study in Rats. Biomed Res Int..

[37] Gredes T, Gedrange T, Hinuber C, Gelinsky M, Kunert-Keil C (2015). Histological and molecular-biological analyses of poly(3-hydroxybutyrate) (PHB) patches for enhancement of bone regeneration. Ann Anat..

[38] Gredes T, Kunert-Keil C, Dominiak M, Gedrange T, Wrobel-Kwiatkowska M, Szopa J (2010). The influence of biocomposites containing genetically modified flax fibers on gene expression in rat skeletal muscle. Biomed Tech (Berl).

[39] Miura C, Shimizu Y, Imai Y, Mukai T, Yamamoto A, Sano Y (2016). In vivo corrosion behaviour of magnesium alloy in association with surrounding tissue response in rats. Biomed Mater.

[40] Kunert-Keil C, Gredes T, Heinemann F, Dominiak M, Botzenhart U, Gedrange T (2015). Socket augmentation using a commercial collagen-based product--an animal study in pigs. Mater Sci Eng C Mater Biol Appl..

[41] Ashammakhi N, Suuronen R, Tiainen J, Tormala P, Waris T (2003). Spotlight on naturally absorbable osteofixation devices. J Craniofac Surg.

[42] Byun JH, Lee HA, Kim TH, Lee JH, Oh SH (2014). Effect of porous polycaprolactone beads on bone regeneration: preliminary in vitro and in vivo studies. Biomater Res..

[43] Galgut P, Waite I, Smith R (1996). Tissue reactions to biodegradable and non-degradable membranes placed transcutaneously in rats, observed longitudinally over a period of 4 weeks. J Oral Rehabil.

[44] Skorkowska-Telichowska K, Czemplik M, Kulma A, Szopa J (2013). The local treatment and available dressings designed for chronic wounds. J Am Acad Dermatol.

[45] Anderson JM, Rodriguez A, Chang DT (2008). Foreign body reaction to biomaterials. Semin Immunol.

[46] Park JU, Ham J, Kim S, Seo JH, Kim SH, Lee S (2014). Alleviation of capsular formations on silicone implants in rats using biomembrane-mimicking coatings. Acta Biomater.

[47] Galgut P, Pitrola R, Waite I, Doyle C, Smith R (1991). Histological evaluation of biodegradable and non-degradable membranes placed transcutaneously in rats. J Clin Periodontol.

[48] de Tayrac R, Oliva-Lauraire MC, Guiraud I, Henry L, Vert M, Mares P (2007). Long-lasting bioresorbable poly(lactic acid) (PLA94) mesh: a new approach for soft tissue reinforcement based on an experimental pilot study. Int Urogynecol J Pelvic Floor Dysfunct.

[49] Ishii D, Ying TH, Mahara A, Murakami S, Yamaoka T, Lee WK (2009). In vivo tissue response and degradation behavior of PLLA and stereocomplexed PLA nanofibers. Biomacromolecules.

[50] Frank PG, Woodman SE, Park DS, Lisanti MP (2003). Caveolin, caveolae, and endothelial cell function. Arterioscler Thromb Vasc Biol.

[51] Woodman SE, Ashton AW, Schubert W, Lee H, Williams TM, Medina FA (2003). Caveolin-1 knockout mice show an impaired angiogenic response to exogenous stimuli. Am J Pathol.

[52] Rozema FR, De Bruijn WC, Bos RRM (1992). Late tissue response to bone plates and screws of poly(L-lactide) used for fracture fixation of the zygomatic bone.

[53] Fuchs M (2006). Untersuchung zu biodegradablen Osteosynthese-Implantaten.

[54] Williams DF (1987). Biodegradability and toxicity of polymers as adjuvants for parenteral drug delivery sysrems.

[55] Gunatillake PA, Adhikari R (2003). Biodegradable synthetic polymers for tissue engineering. Eur Cell Mater..

[56] Grizzi I, Garreau H, Li S, Vert M (1995). Hydrolytic degradation of devices based on poly(DL-lactic acid) size-dependence. Biomaterials.

[57] Pistner H, Gutwald R, Ordung R, Reuther J, Muhling J (1993). Poly(L-lactide): a long-term degradation study in vivo. I. Biological results. Biomaterials..

[58] von Recum HA, Cleek RL, Eskin SG, Mikos AG (1995). Degradation of polydispersed poly(L-lactic acid) to modulate lactic acid release. Biomaterials.

[59] Thordarson DB, Hurvitz G (2002). PLA screw fixation of Lisfranc injuries. Foot Ankle Int.

[60] Bergsma JE, de Bruijn WC, Rozema FR, Bos RR, Boering G (1995). Late degradation tissue response to poly(L-lactide) bone plates and screws. Biomaterials.

[61] Mainil-Varlet P, Rahn B, Gogolewski S (1997). Long-term in vivo degradation and bone reaction to various polylactides. Biomaterials..

[62] Grayson AC, Voskerician G, Lynn A, Anderson JM, Cima MJ, Langer R (2004). Differential degradation rates in vivo and in vitro of biocompatible poly(lactic acid) and poly(glycolic acid) homo- and co-polymers for a polymeric drug-delivery microchip. J Biomater Sci Polym Ed.

[63] Qu XH, Wu Q, Zhang KY, Chen GQ (2006). In vivo studies of poly(3-hydroxybutyrate-co-3-hydroxyhexanoate) based polymers: biodegradation and tissue reactions. Biomaterials.

[64] Jeong SI, Kim BS, Kang SW, Kwon JH, Lee YM, Kim SH (2004). In vivo biocompatibilty and degradation behavior of elastic poly(L-lactide-co-epsilon-caprolactone) scaffolds. Biomaterials.

[65] Woodward SC, Brewer PS, Moatamed F, Schindler A, Pitt CG (1985). The intracellular degradation of poly(epsilon-caprolactone). J Biomed Mater Res.

[66] Sun H, Mei L, Song C, Cui X, Wang P (2006). The in vivo degradation, absorption and excretion of PCL-based implant. Biomaterials.

[67] Lam CX, Savalani MM, Teoh SH, Hutmacher DW (2008). Dynamics of in vitro polymer degradation of polycaprolactone-based scaffolds: accelerated versus simulated physiological conditions. Biomed Mater.

[68] Ng KW, Achuth HN, Moochhala S, Lim TC, Hutmacher DW (2007). In vivo evaluation of an ultra-thin polycaprolactone film as a wound dressing. J Biomater Sci Polym Ed.

[69] Choy DK, Nga VD, Lim J, Lu J, Chou N, Yeo TT (2013). Brain tissue interaction with three-dimensional, honeycomb polycaprolactone-based scaffolds designed for cranial reconstruction following traumatic brain injury. Tissue Eng Part A.

[70] Bernards DA, Bhisitkul RB, Wynn P, Steedman MR, Lee OT, Wong F (2013). Ocular biocompatibility and structural integrity of micro- and nanostructured poly(caprolactone) films. J Ocul Pharmacol Ther.

